# Voice-Based Assessment of Extrapyramidal Symptoms Using Deep Learning

**DOI:** 10.3390/s25164968

**Published:** 2025-08-11

**Authors:** Erandhi M. Liyanage, Kun-Chan Lan, Quang Ha, Sai Ho Ling

**Affiliations:** 1 School of Electrical and Data Engineering, University of Technology Sydney, 15 Broadway, Ultimo, Sydney, NSW 2007, Australia; quang.ha@uts.edu.au; 2 Department of Computer Science and Information Engineering, National Cheng Kung University, 1, Dasyue Rd, East District, Tainan City 701, Taiwan; 9602016@gs.ncku.edu.tw

**Keywords:** deep learning, extrapyramidal symptoms, voice diagnostics, DenseNet, fundamental frequency, chroma, spectral contrast, Parkinsonism

## Abstract

Extrapyramidal symptoms encompass features of Parkinsonism, including bradykinesia, cogwheel rigidity, and resting tremors, which contribute to motor impairments hindering handwriting and speech. In this study, we analyzed voice data captured using a voice sensor setup from 94 patients exhibiting varying levels of EPS and 30 unaffected controls. Each participant provided 13 recordings of repeated vowel and consonant sounds. The Drug-Induced Extrapyramidal Side Effect Scale and Glasgow Antipsychotic Side Effect Scales were used when grading patients into mild, moderate, and severe extrapyramidal symptoms, both administered by trained clinicians. To develop an objective assessment tool, we employed a transfer learning approach using a DenseNet architecture for feature extraction and classification. Its architecture enables the hierarchical concatenation of features at each layer. In this study, we identified that key acoustic features, MFCC, chroma, and spectral contrast vary significantly with the severity of extrapyramidal symptoms. Based on these findings, we developed a DenseNet-based model capable of predicting extrapyramidal symptoms from voice data. This model can classify with an accuracy of 81.9% and a precision of 82.0%. To the best of our knowledge, this is the first study to introduce a voice-based model for assessing the severity of extrapyramidal symptoms.

## 1. Introduction

Extrapyramidal symptoms (EPSs) are drug-induced movement disorders. Common characteristics of EPSs are Akathisia, Dystonia, Tardive Dyskinesia, and Parkinsonism [1]. They are caused by the blocking of the Dopamine 2 receptors (D2Rs), with the intake of antipsychotic drugs (APs). They affect the nigrostriatal dopamine pathway. The severity of extrapyramidal symptoms (EPSs) varies depending on the type of antipsychotic (AP) medication. First-generation antipsychotics (FGAs) primarily exert their effects by strongly binding to dopamine D_2_ receptors (D_2_Rs), which is associated with a higher risk of EPSs. In contrast, second-generation antipsychotics (SGAs) interact with multiple receptor types, including serotonin receptors, and generally have a lower affinity for D_2_R, resulting in a reduced incidence of EPSs [2,3]. The effectiveness and side effect profiles of these medications can vary between individuals, necessitating personalized treatment approaches [2,3].

Psychiatrists who prescribe them cannot predict if the patient will develop EPSs. Generally, drugs are given and the severity of extrapyramidal side effects is monitored by the physician using manual physical examination scales, such as the Drug-Induced Extrapyramidal Pyramidal Symptom Scale (DIEPSS) [4] and the Glasgow Antipsychotic Side Effect Scale (GASS) [5]. The GASS is a self-rating scale (subjective) that is conducted by the patient [5]. The DIEPSS scale is an objective scale, conducted by a trained psychiatrist [4]. Both scales, though useful, can be variable with assessment scores differing between examiners. These scales are designed to measure the Parkinsonism symptoms that AP medication causes, such as bradykinesia, rigidity, resting tremor, and postural instability [6]. Drug-induced Parkinsonism (DIP) accounts for 20–40% of the main symptoms. These affect the voice, handwriting, and movement of the patient.

Patients with Parkinsonism have an abnormal pitch, a hoarse or “breathy”, strained voice with a resting tremor [7]. These patients have difficulty pronouncing consonants. Changes in loudness are especially seen when speaking continuously [8]. These voice changes are due to rigid laryngeal muscle, bradykinesia, and resting tremor [6,9]. Parkinsonism voice changes have been researched widely while DIP, caused by AP medications, has seen limited research. One paper showed similar voice changes in DIP and PD patients, with a slower articulation rate, increasing pauses, and shorter utterances with the intake of AP medications [10]. To represent voice changes quantitatively, advanced features should be measured such as Mel-Frequency Cepstral Coefficient (MFCC), Spectral Contrast, and Chroma features.

Mel-Frequency Cepstral Coefficients (MFCCs), Chroma features, and Spectral Contrast are widely used to capture the energy variations in voice signals. MFCCs represent the shortterm power spectrum of sound, effectively modeling the configuration of the vocal tract and providing critical frequency and temporal information [11]. Chroma features reflect the distribution of spectral energy across the 12 fundamental frequency (Fo) classes, capturing harmonic and melodic characteristics that are robust to changes in articulation, dynamics, timbre, and local tempo deviations [12]. Spectral Contrast measures the difference between spectral peaks and valleys across frequency bands, indicating how sound level varies across different frequencies [13]. The features of the voice produced by patients have significant differences compared to healthy controls [14], with the features becoming more prominent with the dosage of the AP [14].

However, some earlier studies have shown that the voice features change with the intake of APs. The amount of research is limited and, as of now, there is no quantitative measure of the extent of voice changes. To show that voice features vary between healthy controls and PD patients, artificial intelligence, machine, and deep learning models are increasingly being used.

Machine learning algorithms have previously been used to extract features, such as jitter, fundamental frequency (*F*_0_), and shimmer [15]. Also, there are newer wavelengthbased approaches that can detect Fo accurately [16]. For traditional deep learning models such as CNN, training is required. Usually, medical data are limited, and training such a model would not be efficient with a small dataset. Transfer learning from pre-trained models, such as Inception V3 and DenseNet121, allows for fine-tuning for specific voice classification tasks, producing high-performance models [17].

Contributions: There remains limited research on how voice changes correlate specifically with EPS severity, as induced by antipsychotic medications. This study seeks to fill this gap by focusing on voice data as a potential early indicator of EPS. The contributions of this research are as follows:

1. We offer a comprehensive summary of feature changes with the severity of EPS. The significant differences between the nonmedicated group increase as extrapyramidal symptoms worsen. A quantitative measure of the extent of the MFCC, spectral contract, and chroma characteristics is determined in correlation with the severity of the EPS.
2. We propose a novel model capable of accurately predicting the severity of extrapyramidal symptoms (EPSs). Utilizing a transfer learning approach, we fine-tuned the final dense layers of a pre-trained DenseNet architecture to enhance prediction accuracy. To our knowledge, this is the first DenseNet-based model designed to predict EPS severity using non-invasive techniques, potentially reducing reliance on assessments by trained clinicians.

## 2. Materials and Methods

**Figure 1:**
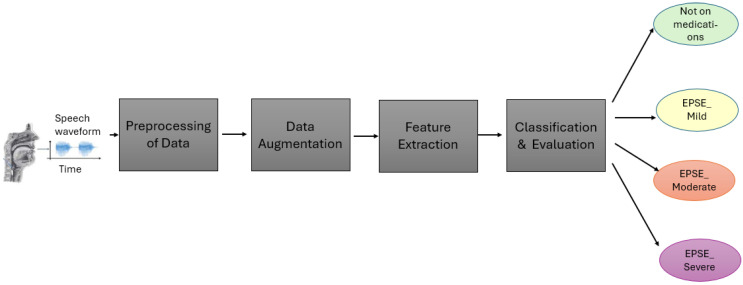
The flow from the dataset, from the classification of the dataset, to how it enters the model and the final classification output.

The voice data collected (Figure 1) were preprocessed to a time frequency representation of sound before being used for feature extraction and training the model. After training, the model produced final classifications into four classes, “Not on antipsychotics”, “Mild”, “Moderate” and “Severe” (Figure 1).

### 2.1. Dataset

The dataset utilized in this study was collected from a psychiatric center in Taiwan (Figure 1). A total of 94 patients exhibiting Parkinsonism symptoms were recruited, all of whom were undergoing antipsychotic (AP) medication treatment. Comprehensive medication histories were collected for each participant. However, the dosage and type of medication were not kept constant between the patients. An approximately equal number of women and men (53 males and 41 females) were recruited between the ages of 21–62 to correct age and gender bias. The severity of extrapyramidal symptoms (EPSs) was assessed using objective measures such as The Drug-Induced Extrapyramidal Symptom Scale (DIEPSS).

Additionally, 30 participants (15 males and 15 females between the ages 20–62) not receiving antipsychotic medications were recruited. All participants were instructed to articulate specific vocal elements, including the vowels “a”, “e”, “I”, “o”, and “u”; the Arabic alphabet; six Taiwanese sentences transcribed in Pinyin; and full sentences in Taiwanese. Each participant provided 13 voice recordings in MP3 format. The recordings were conducted using a mobile device. Only those who completed all 13 recordings were included in the study. With a m = 13 (repeated recordings per person), the effect size was 0.3, intra-person correlation was 0.2, significance level was 0.05, and the desired power was 0.8. The number of participants (n) was calculated using Generalized Estimating Equations (GEEs), with n = 74. The severity of extrapyramidal symptoms (EPSs) was assessed using objective and subjective measures. A trained clinician administered the Drug-Induced Extrapyramidal Symptoms Scale (DIEPSS), which evaluates eight individual items: gait, bradykinesia, sialorrhea, rigidity, tremor, akathisia, dystonia, and dyskinesia—each rated on a scale from 0 (normal) to 4 (severe).

For classification of EPSE severity, the DIEPSS scale was employed by a trained doctor, comprising 8 questions based on symptoms of drug-induced movement disorders, such as gait, bradykinesia, tremor, etc. A score between 0 and 4 was given, along with a global average score [4]. Patients were also asked to self-assess using the GASS scale, which included 16 questions related to the side effects of AP, such as weight gain, dry mouth, and extrapyramidal symptoms [5]. Patients rate this on a frequency scale from 0 to 3, with 0 = never and 3 = most of the time [5]. An overall assessment of the EPS based on these two scales was conducted by an expert. This assessment was also checked along with the GASS and DIEPSS scores, before classifying patients into mild, moderate, and severe classes. Participants not on medications were labeled as not in the antipsychotic group. The individual files were labeled with the participant ID and sample number, such as 00001_01_01.

### 2.2. Preprocessing of Data

All audio recordings were processed using Praat software version 6.2.23. Given that the duration of identical vowels and sentences varied among participants, each recording was manually segmented into 10 s intervals using Praat’s segmentation tools. Following segmentation, the audio files were converted to WAV format to ensure compatibility with subsequent analysis procedures.

For spectrogram visualization and quantitative acoustic analysis, the WAV file was converted into a time-frequency representation using the Short-Time Fourier Transform. For each class, the average Root Mean Square (RMS), mean amplitude, Fundamental Frequency (*F*_0_), Jitter, Shimmer, Amplitude Tremor, and Frequency Tremor (equations above) were calculated. The significant difference between each group was calculated using ANOVA and Tukey’s HSD (Honestly Significant Difference).

### 2.3. Data Augmentation

To enhance the diversity of the dataset, data augmentation techniques were employed. Following preprocessing, random noise—such as chatter and static—was injected into the waveforms to simulate real-world acoustic variations, thereby improving the model’s robustness to noisy data. Additionally, the waveforms were slightly shifted in time, allowing the model to learn temporal variances. The playback speed was artificially altered without changing *F*_0_, introducing variability in speech rate while preserving tonal characteristics. Importantly, *F*_0_, amplitude, and frequency were maintained constant during these augmentations, as alterations in these features can occur due to antipsychotic medication intake and are critical for accurate extrapyramidal symptom (EPS) assessment. After augmentation, the waveforms were transformed into Mel Spectrograms for feature extraction.

### 2.4. Feature Extraction

The Mel Spectrogram generated in the previous phase was input into our DenseNet model for feature extraction. After feature selection, three dominant features were analyzed: MFCCs, Mel Spectrograms (1–40), chroma features (1–12), and spectral contrast features (1–7). These features represent harmonic, spectral, and amplitude characteristics across frequencies, respectively. All four groups—mild, moderate, severe EPS, and not on medication—were processed separately for feature extraction. The extracted features were saved in Excel CSV format. To analyze the extracted features, the relationships between each pair of features were determined using Pearson’s correlation coefficient. Correlation matrices were calculated for all features within each group and visualized using heatmaps. A Student’s t-test was performed on the raw (non-normalized) feature values, comparing the “not on antipsychotic” group with each of the other three groups. *p*-values were calculated, and features with *p*-values less than 0.05 were identified as showing statistically significant differences.

### 2.5. Model Architecture

**Figure 2:**
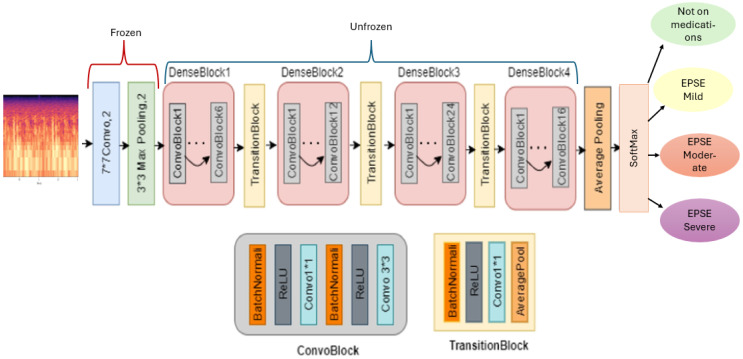
The architecture of the DenseNet121 model that is used for feature extraction and classification.

The architecture of the model consists of the DenseNet backbone (Figure 2). This model shows 4 dense blocks and layers that are frozen and trainable, allowing for transfer learning. The final softmax sorts the multiclass classification into 4 classes. To train the model, an epoch of 50 and a learning rate of 0.0001 were employed (Table 1).

Our model employs transfer learning to enhance classification accuracy. Features are extracted from the input Mel Spectrogram using a pre-trained DenseNet architecture. In this setup, the top 2D convolutional, batch normalization, activation, and pooling layers of the DenseNet are frozen to retain their learned representations, while the remaining four dense blocks are unfrozen and trainable. This approach allows the model to leverage preexisting knowledge while adapting to the specific characteristics of our dataset (Figure 2). The input to the model is a two-channel Mel Spectrogram. Each dense block within the DenseNet comprises multiple bottleneck layers and a transition layer [17].

The dense blocks from 1 to 4 each have 2 convolutional blocks with the first convolutional block having 1 layer and the second convolutional block having 6, 12, 24 (Figure 2), and 16 layers, respectively [17]. To enhance the model’s robustness and prevent overfitting, random dropout is applied during training. Dense blocks facilitate hierarchical learning by concatenating the outputs of all preceding layers, promoting efficient feature reuse and improved gradient flow [17]. This dense connectivity enables the model to construct a more comprehensive representation of the input features, ultimately leading to improved classification performance. A training rate of 0.0001, batch size of 16, epoch of 20, and a scheduler of 3 were used to train the model (Table 1).

### 2.6. Classification and Evaluation

The model utilizes the softmax function to optimize classification after flattening the output layer (Figure 2). It is trained in four classes: “Mild Extrapyramidal Symptoms (Mild EPS)”, “Moderate Extrapyramidal Symptoms (Moderate EPS)”, “Severe Extrapyramidal Symptoms (Severe EPS)”, and “Not on Antipsychotic” medication groups. During training, the model adjusts its weights and biases to minimize the difference between predicted and actual outcomes. A K fold cross-validation of 5 per total number of samples was used to evaluate the robustness of the model (Table 1). The final metrics such as weighted averages of the F1 score, recall, precision, and accuracy are calculated from the average of all 5 folds. Gradient weighted Class Activation Mapping (Grad-CAM) was used to generate class-specific heatmaps.

## 3. Results

**Figure 3:**
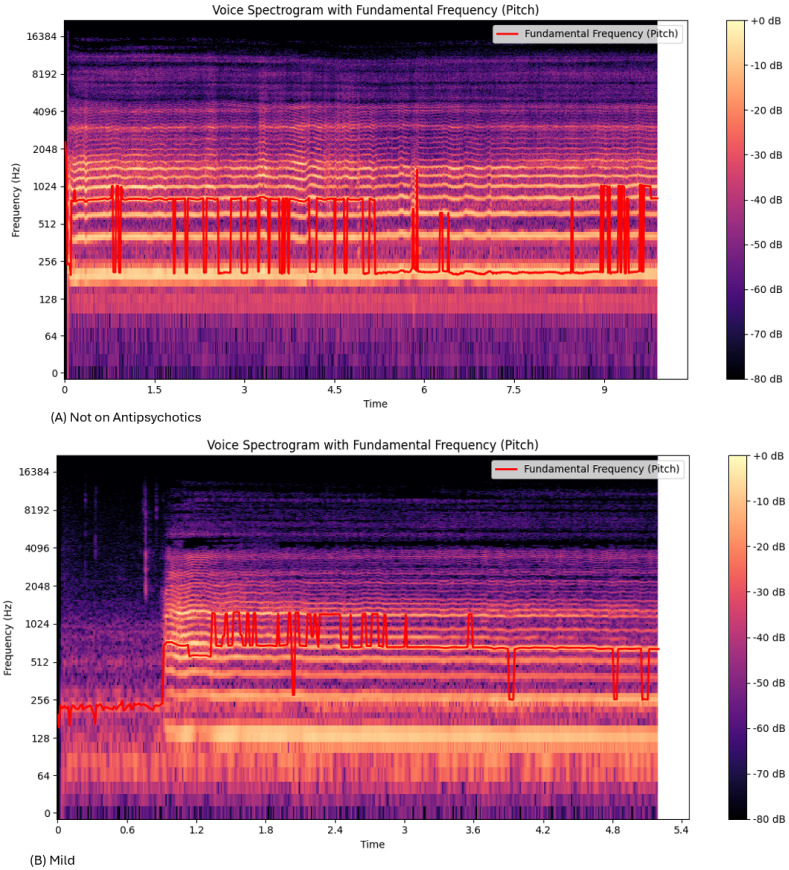

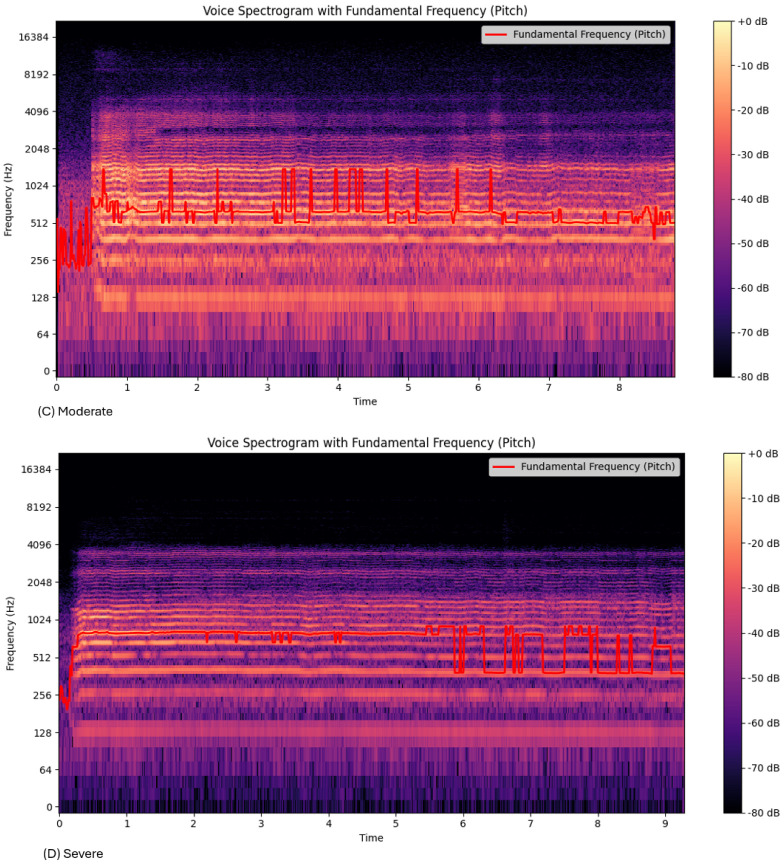
The Mel spectrogram from a single WAV file when repeating the vowel “a”. The voice Mel spectrogram of the four classes: (**A**) not on antipsychotics, (**B**) mild, (**C**) moderate and (**D**) severe.

The frequency, fundamental frequency, energy level and amplitude vary with the severity of extrapyramidal symptoms. Each 10 s voice sample was represented as a timefrequency representation (Figure 3). Figure 3A shows one voice recording of 10 s spectrogram for a patient not on antipsychotics, while Figure 3B shows one from the mild group. For each class from the total sample, the average Root Mean Square (RMS), mean amplitude, fundamental frequency (*F*_0_), jitter, shimmer, amplitude tremor, and frequency tremor (equations above) are represented as averages (Table 2). The significant difference between each group, calculated using ANOVA and Tukey’s HSD (Honestly Significant Difference), is shown (Table 3).

### 3.1. Frequency, Fundamental Frequency (*F*_0_), and Amplitude of the EPS Voice

We assessed changes in voice with the severity of EPSs. As EPSs are caused by antipsychotic drugs, *F*_0_, frequency, and amplitude vary between medicated versus non medicated individuals. The Mel spectrogram from the not on medication group (Figure 3) had a more varying *F*_0_ compared to the EPS groups. The range of the *F*_0_ decreases with severity. The “Not on Antipsychotic”, “Mild”, “Moderate” and “Severe” groups had a max *F*_0_ of 2198.52 Hz, 2079.49 Hz, 169.610 Hz, and 162.00 Hz, respectively (Table 2). There was a significant difference in the mean *F*_0_ between the “Not on Antipsychotics” group versus the other three groups (Table 3).

A resting tremor is observed in patients with extrapyramidal symptoms [6]. The amplitude tremor, which represents the variation in sound intensity, was increased between the non-medicated group versus the medicated group, with the Amplitude Tremor (RMS std) increasing with the severity (Table 2). There was a significant difference between the “Not on Antipsychotic” and “Moderate” group. Also, between the “Not on Antipsychotic” and “Severe” groups (Table 3).

The Frequency Tremor or the oscillation in *F*_0_ was decreased from the “Not on Antipsychotic”, to “Mild”, “Moderate” and “Severe” groups, with frequency tremors of 154.25, 126.63, 116.21 and 109.39, respectively (Table 2). There was a significant difference (p<0.005) between the “Not on Antipsychotic” group and the “Mild”, “Moderate”, and “Severe” groups (Table 3).

### 3.2. Characteristic Features of the Voice

**Figure 4:**
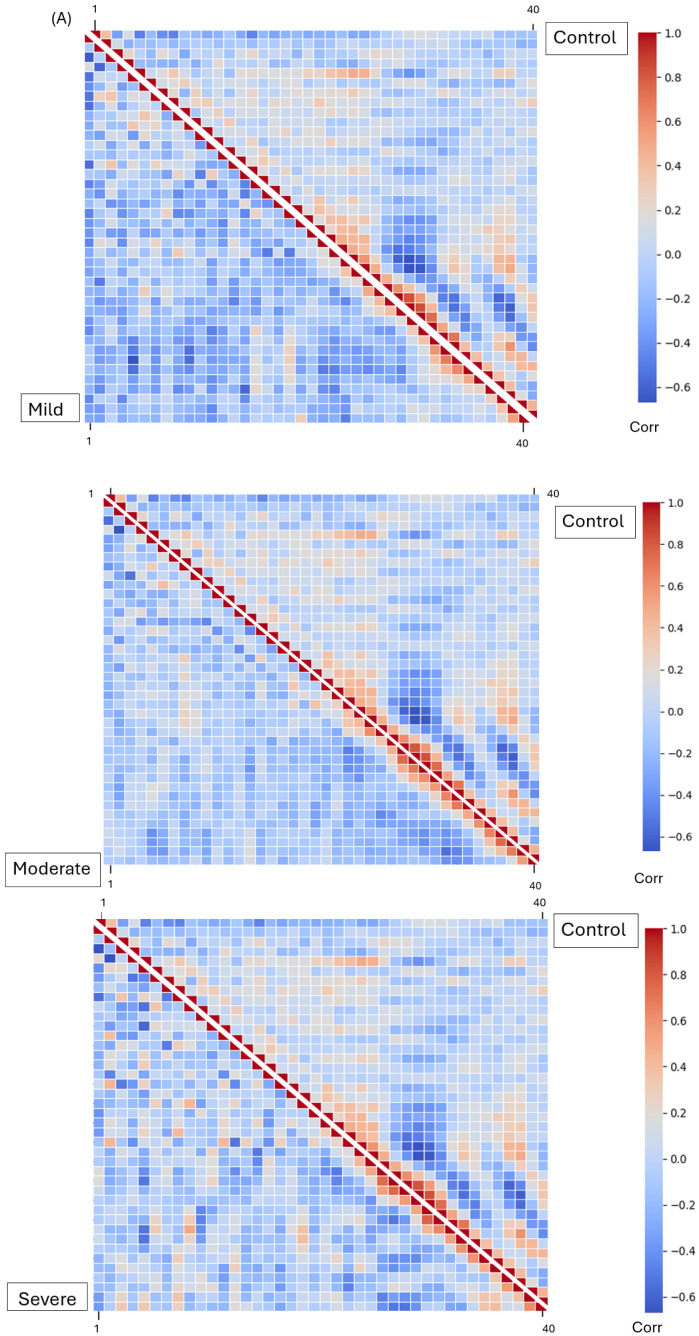

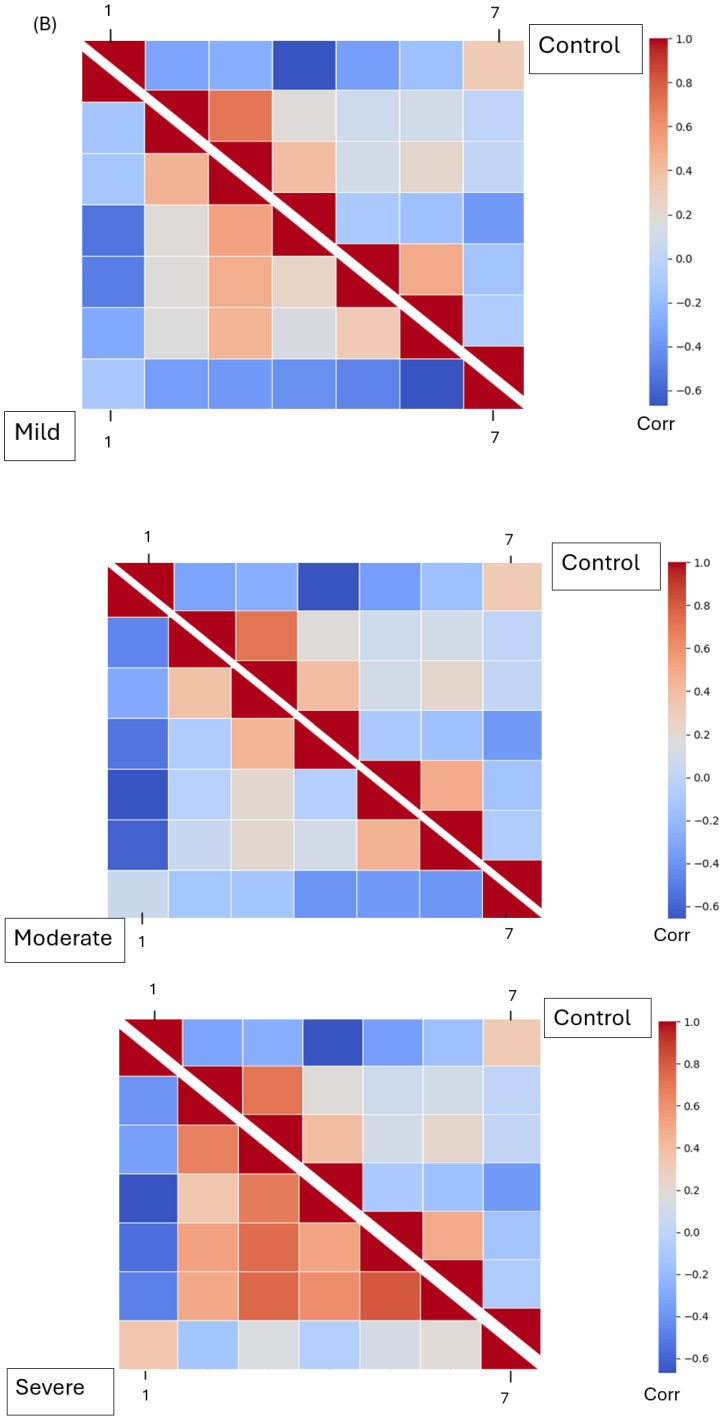

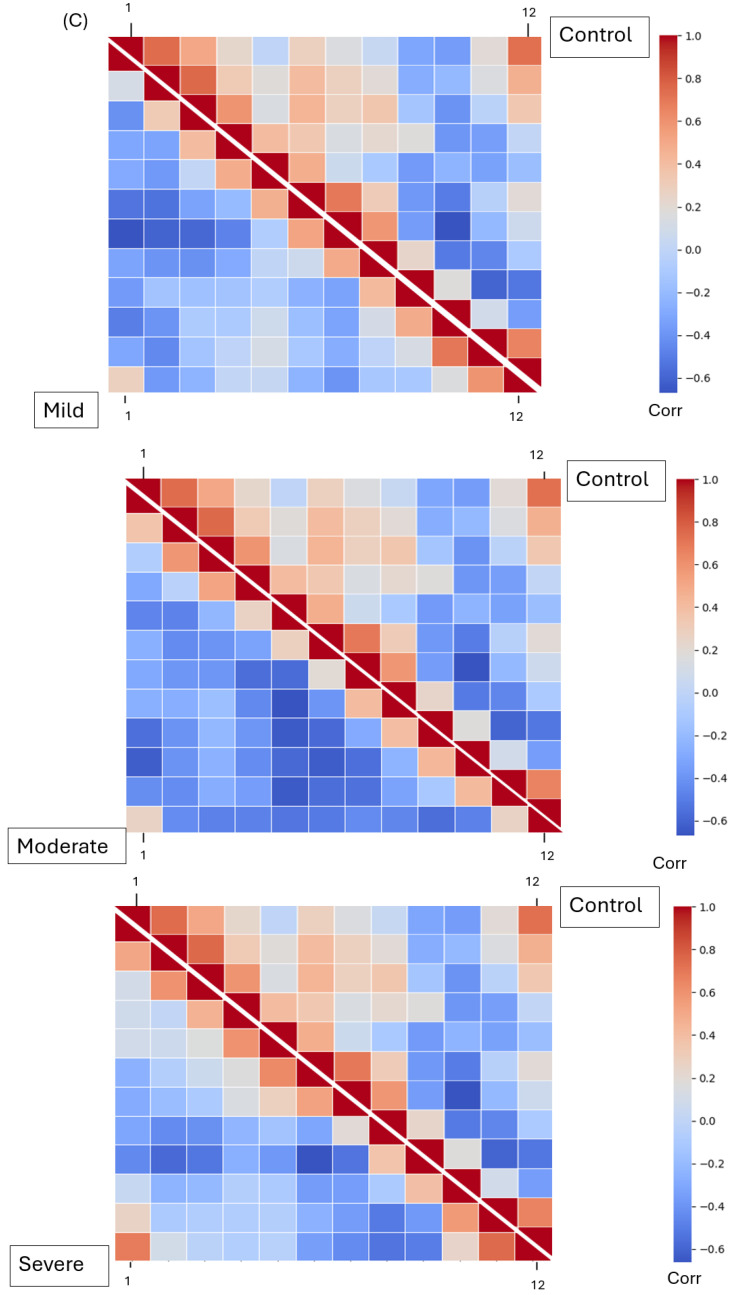
(**A**) The relative correlation coefficients for the control group “Not on Antipsychotics”, compared with EPS severities of “Mild”, “Moderate”, and “Severe” for the MFCC feature. (**B**) The correlation coefficients for the control group “Not on Antipsychotics” were also compared with the EPS severity “Mild”, “Moderate”, and “Severe” for the Spectral Chroma feature. (**C**) The relative correlation coefficients for the control group “Not on Antipsychotics” were also compared with the EPS severity “Mild”, “Moderate”, and “Severe” for the Chroma feature.

We created heat maps of the correlation coefficient matrices of features extracted from the voice recording. The not on antipsychotics group at the top right side on each image is compared to the EPS mild, moderate, and severe (bottom left). Three principal voice features selected after feature extraction are displayed. The correlation coefficient heat map for Mel Frequency Cepstrum Coefficient (MFCC) is plotted as an average per group (Figure 4A). The relative correlation coefficient heat map for the Spectral Contrast feature is presented as an average per group (Figure 4B). The heat map of relative correlation coefficient for the Chroma features was plotted as the average per group (Figure 4C). A mirror image of the control group is shown at the top right to compare significant pattern differences with the three other groups. The T-test (*p* values) was used to calculate between group A (“Not on Antipsychotic”), group B (“Mild”), group C (“Moderate”), and group D (“Severe”) from the heatmaps (Table 4), showing significantly (p<0.001) high MFCC, Chroma and Spectral Contrast features.

Features represent voice changes more accurately and can be used to give a quantitative analysis. Figure 4a above shows that the heatmap for the MFCC for the group “Not on antipsychotics”, compared to the groups on antipsychotic medications, had 10 MFCC features that were significantly different (p<0.05), with MFCC5, (t=5.72,p<0.001), MFCC7 (t=8.44,p<0.001), MFCC10 (t=4.53,p<0.001), MFCC12 (t=5.45,p<0.001), MFCC15 (t=3.58,p<0.001), MFCC24 (t=2.46,p<0.014), MFCC25 (t=2.23,p<0.026), MFCC26 (t=3.77,p<0.001) and MFCC37 (t=3.19,p<0.001) significantly decreased in the severe EPSE groups. Only the features that were significantly different within all three classes are shown here. MFCC4 was the only one that was increased with a T statistic between −8 and −2 (Table 4). The other 9 MFCCs have positive T statistics between 2 and 9.

The chroma features or the phonetic $F_{0}$ were also less. With six chroma features decreased in the mild, moderate, and severe groups compared to the not-on-medication group (Figure 4c). The T statistics ranged from 2 to (p<0.05), including chroma 2 (t=2.74,p<0.001), chroma 3 (t=4.08,p<0.001), chroma 4 (t=4.10,p<0.001), chroma 5 (t=2.78,p<0.005), chroma 6 (t=1.96,p<0.05) and chroma (t=2.83,p<0.05) (Table 4). These were all lower in mild, moderate, and severe groups in comparison to the non-AP group.

The amplitude of or the change in sound level with frequency was higher in the severe group compared to the other three groups. The chroma features increase with the severity (Figure 4b), as with spec contrast 1 (t=−2.573,p=0.010,t=−4924,p<0.001,t=−4191,p<0.001) and spec contrast 3 (t=−4.016,p<0.001,t=−8.135,p<0.001,t=−4.191,p=0.019) (Table 4). This was not the case in the antipsychotics group compared to EPS mild, moderate, and severe groups.

### 3.3. Voice as a Prediction Model

**Figure 5:**
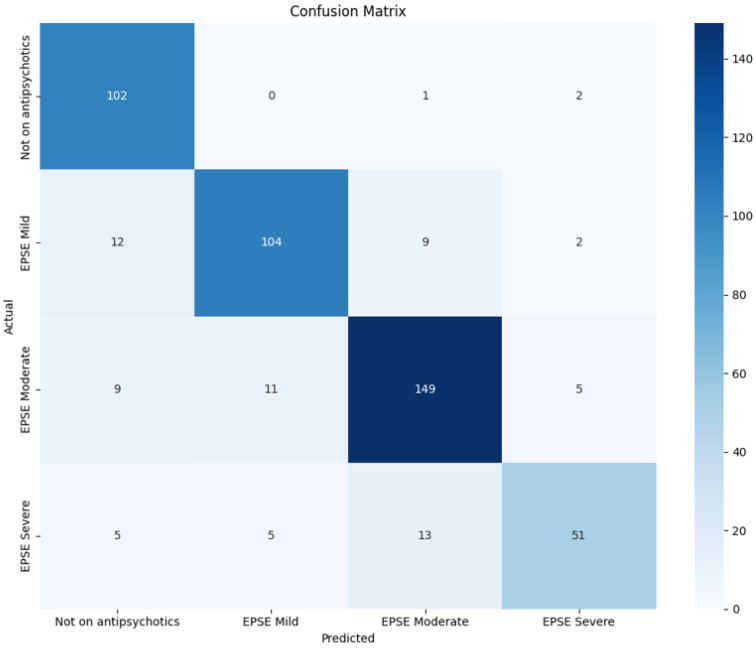
The confusion matrix for the model DenseNet with data augmentation.

The average evaluation matrix from the 5-fold cross-validation for the DenseNet121 and the ResNet152 is displayed (Table 5). The same experimental conditions and confusion matrix are used for both models (Figure 5).

The DenseNet model with 121 layers performs almost twice as well as the ResNet152 model, in all evaluation matrices (accuracy, precision, recall and f1 score). The accuracy, precision, recall and F1 score for the DenseNet121 model are 81.9%, 82.0%, 81.9% and 81.8%, respectively (Table 5). The true positive for the predicted value was higher than the false negative and false positives (Figure 5).

**Figure 6:**
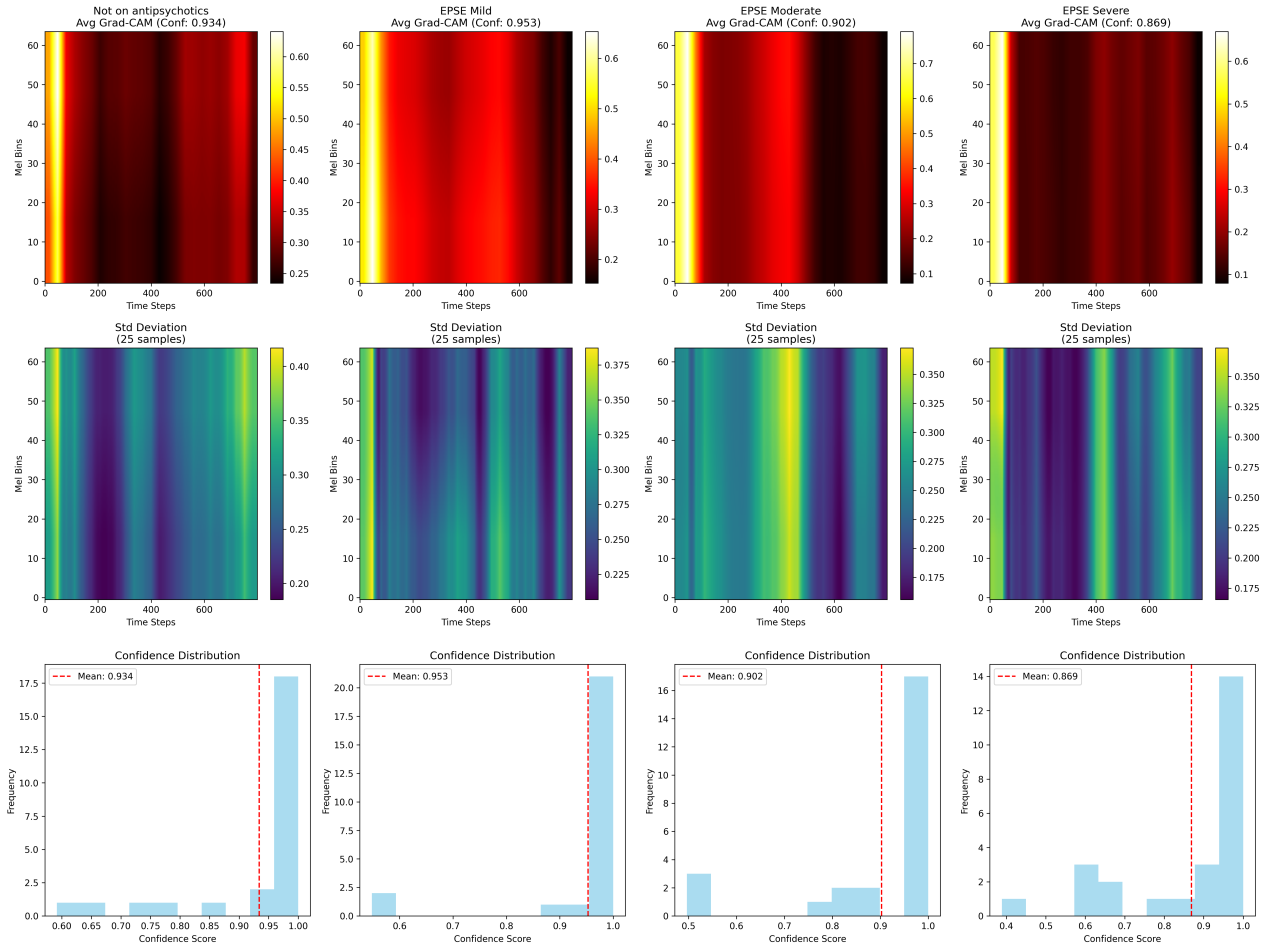
The visualizations show the averaged Grad-CAM heatmaps across all the 5 folds for each EPSE severity level. The standard deviation per class and the confidence for each class.

Grad-CAM was utilized to visualise the area of the image that the DenseNET121 focused on the most when making the classification decision (Figure 6).

The Not on Antipsychotics group had strong activation across most frequency bands (strong red/yellow activation), a higher activation in upper Mel bins and a consistent intensity across time (Figure 6), while the EPSE mild group had slightly reduced activation. With more concentrated activation bands, the Moderate EPSE group showed further noticeable reduction in activation intensity (darker regions). The EPSE Severe group, in comparison, had much more darker regions and concentrated activation in specific frequency bands (Figure 6). Furthermore, there were two peaks in all groups except the EPSE severe which had three peaks. The confidences of the Grad-CAM for not on antipsychotic, mild, moderate and severe groups were 93.4%, 95.3%, 90.2% and 86.9%, respectively.

## 4. Discussion

Extrapyramidal symptoms (EPSs) are side effects resulting from the intake of antipsychotic medications [4]. The extent or severity of EPS is monitored by psychiatrists through physical examinations of the patient. Both subjective scales, such as the Glasgow Antipsychotic Side-effect Scale (GASS) [5], and objective scales, like the Drug-Induced Extrapyramidal Symptoms Scale (DIEPSS) [4], are utilized to assess these symptoms. However, this method of monitoring side effects is time-consuming and susceptible to human error. Voice alterations are among the characteristics that can change due to the Parkinsonism symptoms associated with EPS. Patients with Parkinsonism often exhibit differences in phonetics, amplitude, and $F_{0}$ [6,9]. By employing advanced feature extraction techniques, these vocal changes can be analyzed to identify the severity of the disease [14].

The Mel Spectrograms (Figure 3) show variations in the $F_{0}$ oscillation between mild, moderate, and severe groups. The range of $F_{0}$ for the “Not on Antipsychotic” group (Table 2) was much higher than the “Severe” group (Table 3). This could be due to the effect of bradykinesia and result in more monotonous voice characteristics in the patients [6].

The amplitude tremor, or the sound level over time, increased with the severity of EPSs. This is a characteristic of PD patients. Studies have shown that PD patients often exhibit rhythmic amplitude fluctuations [8,18]. The variation in amplitude over time may be due to the reduced ability of the EPS brain to compensate for tremor [18], increasing the resting tremor and the fluctuations in sound levels [6,9].

The frequency tremor, or the range of the $F_{0}$ (Table 2), shows a decrease from the nonmedicated group to the severe EPS group. Figure 3 and Table 2 show that the frequency lowered with EPS severity, as represented by the increased purple in Figure 3d. This correlated with the lower $F_{0}$ range (Table 2). Previous research has shown similar findings, showing that the tremor frequency drops with EPS severity [19]. This may be due to the rigidity and bradykinesia, causing increasing stiffness and lower frequency.

Features extracted from voice recordings of patients with mild, moderate, and severe symptoms exhibit varying levels of expression. MFCCs (Mel-frequency cepstral coefficients) capture the short-term spectral properties of sound, representing the number of vibrations at each frequency [18]. The numbers represent each coefficient extracted from the audio signal, each capturing the different spectral characteristics of sound. The MFCC1-2 captures the broad energy distribution [20], MFCC3-13, containing finer details of the spectral shape. The MFCC 14-35 represents higher frequency variations, which is used for speaker identification/emotional recognition [20]. Ten MFCC features showed significant differences between the “Not on Antipsychotic” group and the three groups receiving medication. Specifically, MFCC5, MFCC7, MFCC10, MFCC12, MFCC15, MFCC24, MFCC25, MFCC26, and MFCC37 were significantly decreased in the severe EPS group (Figure 4). This decrease in energy could be due to the monotonic or restricted speech patterns seen in patients with EPS. The bradykinesia slows down the movement in the larynx and the articulatory muscles, resulting in a more monotonous voice. Additionally, MFCC4, MFCC5, MFCC10, and MFCC12 have previously been reported to differ significantly between the placebo and medicated groups [14]. However, these four features did not increase with higher olanzapine-equivalent doses. MFCC1, MFCC8, and MFCC12 increased with rising doses of antipsychotics (AP) [14]. Changes in voice frequency can be represented by MFCCs, and vice versa. However, frequency has shown inconsistent results in relation to symptoms of Parkinsonism. For example, schizophrenia patients on antipsychotics exhibited higher voice frequencies that correlated with EPS severity [21]. In contrast, another study found that Parkinson’s disease (PD) patients showed reduced fundamental frequency, intensity, and harmonic-to-noise ratio, with these changes remaining consistent regardless of disease duration or severity [22].

Spectral contrast represents the amplitude of the energy spectrum between peaks and valleys of an audio signal. The following bands 1, 2, 3, 4, 5, 6 and 7, of the spectral contrast, capture the lowest, low-mid, mid, upper mid-range, high, very high, and highest frequency, respectively [23]. Figure 4 shows spectral contrast was found to be higher in patients with extrapyramidal symptoms (EPSs), increasing with severity. In particular, spectral contrast 1 (t=−4.19,p<0.001) and spectral contrast 3 (t=−2.35,p=0.018) showed significant differences. A study conducted nearly 20 years ago supports these findings, observing that patients with drug-induced Parkinsonism exhibited characteristic rabbit syndrome, which included louder voices, decreased frequency, and lower jaw tremors [24,25].

Chroma features capture the harmonic and melodic characteristics of audio signals in the 12-pitch classes. Chroma 1, is for the C pitch, chroma 2 is for C, Chroma 3 is for the D pitch, chroma 4 is for the D pitch class, chroma 5 is for energy in the E pitch, chroma 6 is for F pitch, 7 is for F# pitch, 8 is for G pitch, 9 is for G pitch, 10 is for A pitch, chroma 11 is for A pitch and chroma 12 is for B pitch. In our study, the energy distribution in $F_{0}$ decreased, as indicated by six chroma features, Chroma 2, 3, 4, 5, 6, and 10, which were all lower in the mild, moderate, and severe EPS groups compared to the non-antipsychotic group (Figure 4). This reduction in pitch could be due to the stiffness in the vocal muscles that restricts the ability to modulate pitch. Also, a hoarse and breathy pitch was observed in patients, aligning with previous findings [7].

The voice acoustic features correlate with the Grad-CAM spectrogram results. The frequency tremor emerging early between the Mild and Not on Antipsychotics (p=0), and the significant amplitude tremor in the moderate/severe groups compared to the not on antipsychotics group, is confirmation of the Grad-CAM frequency band shifts (Figure 6). The decreased spectral contrast observed (Figure 4) in the severe group correlates with the reduced Grad-CAM activation intensity (Figure 6). This could be due to the flattening spectral envelope, indicating a loss of clarity and harmonics [26]. There were three peaks in the severe group compared to the other groups that show two peaks (Figure 6). This could be due to the fluctuations in the speech patterns that are seen with the increase in severity [27].

As features vary with the severity of EPSs, a model trained on these features can accurately predict whether patients are experiencing mild, moderate, or severe symptoms. Our model achieves an accuracy, precision, recall, and F1 score of 81.9%, 82.0%, 81.9%, and 81.8% (Table 5), respectively. A K-fold cross-validation of five per total sample was used, instead of per class or subject. This improves the robustness of the model to handle uneven clinical data. Given the limited availability of medical data, we employ a transfer learning approach. The DenseNet121 model is pre-trained on several datasets, including ImageNet, CIFAR-10, CIFAR-100, and Street View House Numbers (SVHN) [17]. The pretrained weights allow the model to recognize a wide range of features from various visuals, including Mel spectrogram images [17]. DenseNet121 connects each layer in an efficient feedforward manner, promoting feature reuse and mitigating the vanishing gradient problem [17]. It outperforms the ResNet model on the same dataset under identical conditions of data augmentation, model training, and ensemble learning. A separate study supports this finding, showing that the DenseNet model with data augmentation achieved an accuracy of 89.5%, compared to ResNet’s 87.3% on music audio feature classification [28]. These results indicate that the DenseNet-based model performs at almost double the accuracy of the ResNet152 model. This could be due to the effective feature reuse that is seen in DenseNet-based models enabling the capturing of fine-grained frequency details [17]. In comparison, the ResNet152-based model relies heavily on the residual connections with no feature reuse.

One limitation of this research is the small dataset size; utilizing a larger dataset would enhance the model’s training efficacy. Since a model’s robustness heavily depends on the quality and diversity of its training data, expanding the dataset could help mitigate overfitting. Employing techniques such as Generative Adversarial Networks (GANs) [29] can be beneficial for generating synthetic data to augment limited datasets. Also, due to this size limitation, five-fold cross-validation could not be used at the participant level; hence, there is a possibility of data leakage between the training and testing groups. While there is a diverse age and gender group, there is, however, a geographical and linguistic limitation.

Although our model achieves an accuracy of 82%, a higher accuracy is desirable for medical diagnoses to ensure reliability. Another weakness of this study is the variable type, dosage, and duration of medication. Furthermore, clinical diagnoses are seldom based on a single modality; incorporating multiple modalities affected by symptoms like resting tremor, cogwheel rigidity, and bradykinesia could provide a more comprehensive assessment of disease progression. To facilitate this as part of our future research, we would develop a multimodal model combining handwriting and voice modalities. This would enable healthcare professionals to make more informed and personalized diagnostic decisions.

## 5. Conclusions

We propose, for the first time, using voice as a clinical assessment for EPSs. To our knowledge, we are the first to confirm that $F_{0}$, tremor frequency, and tremor amplitude significantly vary with the severity of EPS, along with dominant features Chroma, Spectral Contrast, and MFCC. In terms of the frequency tremor, fundamental frequency, and features MFCC, Chroma were significantly lower between the medicated and non-medicated groups, indicating a reduction in the rate of vocal cord vibrations and the ability to modulate pitch. Spectral contrast and amplitude tremor were significantly increased with EPS severity, highlighting the fluctuations in intensity. Based on these biomarkers, we put forward a voice diagnostic model for EPS. Our model can classify with an accuracy and precision of 81.9% and 82.0%, respectively. This voice-based diagnostic tool for EPS has the potential to be used by patients to monitor symptoms and aid psychiatrists in determining EPS severity.

## Figures and Tables

**Table 1:** Training configuration used for the experiments.

Parameter	Value
Epochs	50
Batch Size	16
Learning Rate	0.0001
Optimizer	Adam optimizer
Scheduler	StepLR (step size = 3)
K-Folds	5

StepLR refers to the learning rate scheduler used with a step size of 3.

**Table 2:** Acoustic parameters across different levels of EPS severity.

Parameter	Not on Antipsychotics	Mild	Moderate	Severe
Sample Rate (Hz)	44,100	44,100	44,100	44,100
Frequency Range (Hz)	0.00–22,050.00	0.00–22,050.00	0.00–22,050.00	0.00–22,050.00
Amplitude (RMS)	0.0636	0.0910	0.1680	0.0964
Amplitude (Max)	0.4228	0.5318	0.7237	0.6660
Amplitude (Min)	0.0000	0.0000	0.0000	0.0000
Amplitude (Mean)	0.0479	0.0684	0.1272	0.0620
Fundamental Frequency (*F*_0_) Mean (Hz)	511.99	1165.64	652.75	703.24
Fundamental Frequency (*F*_0_) Max (Hz)	2316.59	1631.91	907.30	909.37
Fundamental Frequency (*F*_0_) Min (Hz)	198.69	215.17	198.65	195.42
Jitter (local)	0.004736	0.003870	0.005770	0.002110
Shimmer (local)	0.050314	0.059745	0.068819	0.082585
Amplitude Tremor (RMS STD)	0.015178	0.029181	0.071910	0.060735
Frequency Tremor (Pitch Derivative STD)	129.13	158.75	42.22	52.46

**Table 3:** Comparison of *p*-value for frequency tremor, amplitude tremor and fundamental frequency.

Group Comparison	Frequency Tremor *p*-Value	Amplitude Tremor *p*-Value	Fundamental Frequency (*F*_0_) *p*-Value
Mild vs. Moderate	0.1587	0	0.55
Mild vs. Severe	0.0277	0.2791	0.3238
Moderate vs. Severe	0.6609	0	0.9123
Mild vs. Not on Antipsychotics	0	0.4022	0
Moderate vs. Not on Antipsychotics	0	0.0005	0
Severe vs. Not on Antipsychotics	0	0.0108	0

All *p*-values are based on statistical comparisons of acoustic tremor and pitch parameters between groups.

**Table 4:** T-statistics and *p*-values for each feature comparing: A (Not on Antipsychotic) vs. B (Mild), A vs. C (Moderate), and B vs. D (Severe).

Feature	Comparison	T-Statistic	*p*-Value
MFCC4	A vs. B	−2.934	$3.4\times 10^{-3}$
	A vs. C	−8.674	$2.43\times 10^{-17}$
	B vs. D	−5.462	$6.92\times 10^{-8}$
MFCC5	A vs. B	6.797	$2.25\times 10^{-11}$
	A vs. C	8.534	$7.41\times 10^{-17}$
	B vs. D	5.716	$1.73\times 10^{-8}$
MFCC7	A vs. B	8.948	$3.07\times 10^{-18}$
	A vs. C	9.496	$2.63\times 10^{-20}$
	B vs. D	8.446	$2.31\times 10^{-16}$
MFCC10	A vs. B	9.650	$8.55\times 10^{-21}$
	A vs. C	5.749	$1.29\times 10^{-8}$
	B vs. D	4.532	$7.07\times 10^{-6}$
MFCC12	A vs. B	8.287	$5.75\times 10^{-16}$
	A vs. C	6.408	$2.55\times 10^{-10}$
	B vs. D	5.455	$7.21\times 10^{-8}$
MFCC15	A vs. B	6.470	$1.82\times 10^{-10}$
	A vs. C	3.759	$1.84\times 10^{-4}$
	B vs. D	3.587	$3.63\times 10^{-4}$
MFCC24	A vs. B	5.299	$1.55\times 10^{-7}$
	A vs. C	5.090	$4.50\times 10^{-7}$
	B vs. D	2.462	$1.41\times 10^{-2}$
MFCC25	A vs. B	5.484	$5.78\times 10^{-8}$
	A vs. C	5.007	$6.84\times 10^{-7}$
	B vs. D	2.225	$2.64\times 10^{-2}$
MFCC26	A vs. B	5.352	$1.17\times 10^{-7}$
	A vs. C	6.466	$1.78\times 10^{-10}$
	B vs. D	3.768	$1.81\times 10^{-4}$
MFCC37	A vs. B	2.227	$2.63\times 10^{-2}$
	A vs. C	3.842	$1.32\times 10^{-4}$
	B vs. D	3.199	$1.5\times 10^{-3}$
Chroma2	A vs. B	3.966	$8.03\times 10^{-5}$
	A vs. C	2.320	$2.06\times 10^{-2}$
	B vs. D	2.739	$6.3\times 10^{-3}$
Chroma3	A vs. B	3.482	$5.28\times 10^{-4}$
	A vs. C	3.209	$1.4\times 10^{-3}$
	B vs. D	4.082	$5.07\times 10^{-5}$
Chroma4	A vs. B	3.674	$2.57\times 10^{-4}$
	A vs. C	4.489	$8.23\times 10^{-6}$
	B vs. D	4.104	$4.63\times 10^{-5}$
Chroma5	A vs. B	3.148	$1.7\times 10^{-3}$
	A vs. C	4.200	$2.98\times 10^{-5}$
	B vs. D	2.782	$5.6\times 10^{-3}$
Chroma6	A vs. B	2.575	$1.02\times 10^{-2}$
	A vs. C	2.156	$3.14\times 10^{-2}$
	B vs. D	1.964	$4.99\times 10^{-2}$
Chroma10	A vs. B	4.091	$4.79\times 10^{-5}$
	A vs. C	4.352	$1.53\times 10^{-5}$
	B vs. D	2.839	$4.7\times 10^{-3}$
Spectral Contrast1	A vs. B	−2.573	$1.03\times 10^{-2}$
	A vs. C	−4.924	$1.04\times 10^{-6}$
	B vs. D	−4.191	$3.19\times 10^{-5}$
Spectral Contrast3	A vs. B	−4.016	$6.54\times 10^{-5}$
	A vs. C	−8.135	$1.63\times 10^{-15}$
	B vs. D	−2.355	$1.89\times 10^{-2}$
Spectral Contrast7	A vs. B	2.690	0.0073
	A vs. C	4.228	2.64 × $10^{-5}$
	B vs. D	4.893	1.28 × $10^{-6}$

T-statistics and *p*-values are derived using independent t-tests.

**Table 5:** Performance comparison of deep learning models on classification task.

Model	Accuracy (%)	Precision (%)	Recall (%)	F1 Score (%)
DenseNet121	81.9	82.0	81.9	81.8
ResNet152	42.80	41.42	42.80	45.95

## Data Availability

The data presented in this study are available on request from the corresponding author.
